# Proteomic Analysis of Lipid Droplets in *Sesamum indicum*

**DOI:** 10.1007/s10930-020-09902-3

**Published:** 2020-05-29

**Authors:** Satoshi Hamada, Akihiro Kishikawa, Motonobu Yoshida

**Affiliations:** 1 Department of Agricultural Science, Kinki University, Nakamachi 3327-204, Nara, 631-8505 Japan; 2 Osaka University of Comprehensive Children Education, 6-4-26 Yusato, Higashisumiyoshi-ku, Osaka, 546-0013 Japan

**Keywords:** *Sesamum indicum*, Lipid droplet formation, Sesame seed, Proteomics, Oleosin modification, Transportation

## Abstract

We attempted to identify the total proteome in sesame lipid droplets. Results from two-dimensional electrophoresis showed 139 protein spots in lipid droplet samples. Each spot was isolated, digested with trypsin, and applied to liquid chromatography–tandem mass spectrometry (Q-Tof Premier). As a result, 103 spots were identified. Although oleosin, caleosin, and steroleosin are known major components of the lipid droplet, many other proteins were also found in the lipid droplet. In addition to the three major proteins, TAG factor protein, glyceraldehyde-3-phosphate dehydrogenase, F_1_ ATPase, 70-kDa heat shock protein, seed maturation protein PM24, and 11S globulin precursor isoforms 3 and 4 were found in the lipid droplet. Three types of oleosins, 15-, 15.5-, and 17-kDa were present in the sesame lipid droplet, and the 15.5-kDa oleosin had high homology with oleosin from *Coffea canephora*. It has been shown by acid phosphatase treatment that oleosin proteins contain phosphate groups. Protein disulfide-isomerase 2 precursor, calreticulin-1, and BiP, which are known as marker proteins of the endoplasmic reticulum, were found as the components of the lipid droplet. Immunoconfocal microscopy was used to show that 11S globulin precursor isoform 3 and 4 were indeed localized in the lipid droplet. The presence of 11S globulin in the lipid droplets suggested a new mechanism for the lipid droplet formation.

## Introduction

**Fig. 1:**
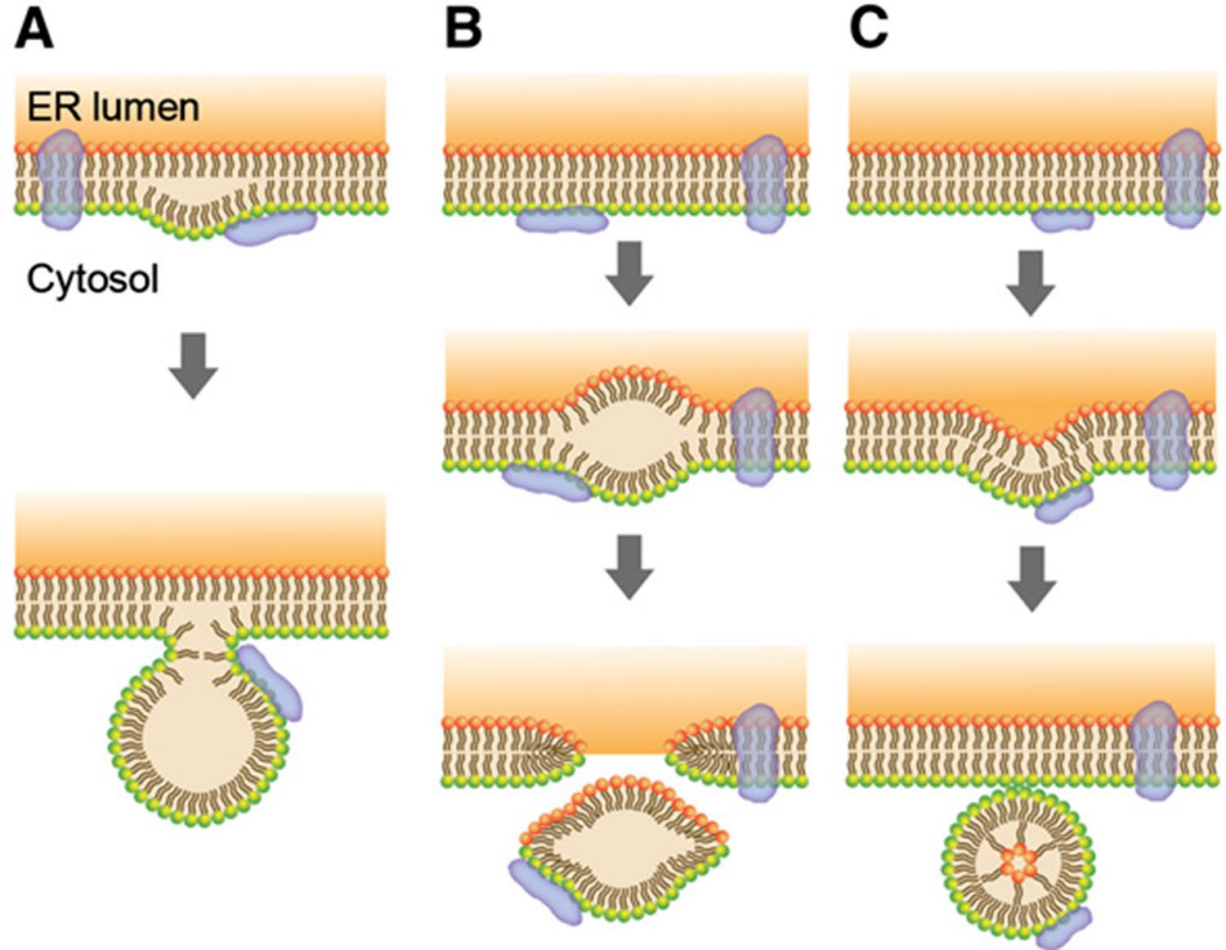
Models of lipid droplet formation. **a** triacylglycerols (TAGs) are deposited between the leaflets of the ER membrane. After reaching a critical size to form a lipid droplet, the TAG core buds off from the ER membrane. Lipid droplets are covered with a monolayer from the leaflet of the cytosol side of the ER membrane. **b** Lipid droplet formation is similar to model (**a**). However, lipid droplets are excised from the ER membrane, and both ER membrane leaflets contribute to the lipid droplet surface monolayer. **c** Inclusion of the TAG core in the membrane vesicle requires rearrangement of the leaflet of lumen side of the ER membrane

The usage of biodiesel and bioethanol instead of petroleum resources has recently come to attention because these fuels have the potential to be less harmful to the environment. Oil crops, including sesame, are used as energy resources as well as crop resources [1]. Among these oil crops, sesame seeds are good energy resources because they contain high amounts of oleic acid, which can be converted to biodiesel with high efficiency. From this point of view, it is important to investigate the components or the formation mechanism of the lipid droplets, the oil storage organelle, of sesame seeds in more detail in order to obtain high productivity from sesame oil. It was shown previously that three proteins are key components of sesame lipid droplets [2, 3]. Oleosin is a major lipid droplet protein, with isoforms with molecular weights of 15-, 15.5-, and 17-kDa. Oleosin proteins function to block the fusion of lipid droplets. In addition, they are known to contain caleosin (molecular weight 27-kDa) that has a calcium-binding motif, and steroleosin (molecular weights 39- and 42-kDa) that retains sterol and NADPH-binding abilities. It has been suggested that these major proteins are synthesized in the endoplasmic reticulum (ER) and then incorporated into the lipid droplets on separation of the lipid droplets from the ER at the time of budding [4]. On the other hand, it has also been suggested that lipid droplet proteins may be transported to the droplet after formation of the droplet [5]. Moreover, there are several hypotheses concerning the formation of lipid droplets (Fig. 1, [6]). (1) Lipid droplets bud off toward the cytosol. (2) Lipid droplets are formed and leave behind a gap in the ER membrane, and afterwards the gap closes rapidly. (3) Lipid droplets form remodeling of the ER-derived bilayer to yield a monolayer covering the droplet. At present, the formation mechanism of lipid droplets and the transportation mechanisms of lipid droplet proteins are unclear. In these circumstances, we focused on the proteomics of lipid droplets to identify the full lipid droplet proteome and clarify the formation mechanism of lipid droplets. In the present study, numerous proteins were found as components of the lipid droplet. Protein disulfide-isomerase 2 precursor, calreticulin-1, and BiP, as a marker protein of the ER, were found in the lipid droplet [7]. Taking these results into consideration, we will discuss the mechanism of the lipid droplet formation.

## Materials and Methods

### Plant Materials

Sesame, *Sesamum indicum*, seeds (Acc. No. 800) were obtained from Toyama University. Toyama University is a sesame stock center in Japan. The Acc. No. of each variety has been registered there. Mature sesame seeds were grown, harvested at a field of Kinki University, and used to prepare lipid droplets.

### Isolation and Purification of Lipid Droplets

Sesame seeds, 0.4 g were immersed for 30 min at 4 °C in 4 ml of 0.6 M sucrose/10 mM Na_2_HPO_4_–KH_2_PO_4_, pH 7.5 (PB) and homogenized using a polytron homogenizer PT2100 (Kinematica AG, Luzern, Switzerland) at 22,000 rpm for 20 s. as described previously [8]. The homogenates were passed through a 40 µm filter. After filtration, the sample was placed at the bottom of a centrifuge tube, and the same volume of medium containing 0.4 M sucrose/PB was layered on top. The samples were centrifuged at 9000×*g* for 20 min. After centrifugation, the top layer including lipid droplets was collected as sample I and placed at the bottom of a fresh centrifuge tube. The same volume of medium containing 0.2 M sucrose/PB was layered on top. After centrifugation at 9000×*g* for 20 min, the top layer including lipid droplets was collected as sample II and placed at the bottom of a fresh centrifuge tube, and the same volume of PB was layered on top. The samples were centrifuged again at 9000×*g* for 20 min. The top layer including lipid droplets was prepared as sample III for focused proteomics analysis. In immunofluorescence experiments, part of sample II was used to examine the localization of 11S globulin a native lipid droplets, and each sample was treated with four kinds of detergent, Triton X-100, Tween 20, SDS or CTAB, at 0.5% final concentrations for 2 h at room temperature (RT).

### SDS-Polyacrylamide Gel Electrophoresis

Samples were subjected to SDS-polyacrylamide gel electrophoresis (PAGE) with 12.5% acrylamide gels using the standard method [9]. After electrophoresis, the gel was stained with 2-D SILVER STAIN**·**II ”DAIICHI” (Daiichi Pure Chemicals, Tokyo, Japan) or Coomassie brilliant blue (CBB).

### Two-Dimensional Electrophoresis

Samples were solubilized with 200 µl of sample buffer containing 8 M urea, 50 mM dithiothreitol (DTT), 2% CHAPS, 0.001% bromophenol blue, 0.2% ampholine pH 3.5–10 (GE Healthcare, Buckinghamshire, UK) and applied onto 11 cm IPG ReadyStrip 3–10 (Bio-Rad) and focused using a PROTEAN^®^ IEF Cell (Bio-Rad). Strips were rehydrated for 12 h at 20 °C in passive mode, and focused at 250 V for 15 min, 8000 V for 1 h, and 8000 V for 35,000 V-h. Before second dimension electrophoresis, strips were kept at RT for 20 min in equilibration buffer I containing 6 M urea, 2% SDS, 20% glycerol, 0.375 M Tris-HCl, pH 8.8, 2% DTT. Next, the strips were kept at RT for 10 min in equilibration buffer II containing 6 M urea, 2% SDS, 20% glycerol, 0.375 M Tris-HCl, pH 8.8, 2.5% iodoacetamide. Second dimension electrophoresis was performed in SDS-PAGE with 12.5% acrylamide, at a constant current of 6 mA. The gels were stained with CBB. A part of sample II was treated with acid phosphatase from wheat germ (230 µg, Nacalai Tesque, Kyoto, Japan) in 1100 µl 10 mM acetate buffer, pH 5.0, 0.1% NP-40 for 14 h at 40 °C. The sample was applied onto 7 cm IPG ReadyStrip 3–10 and analyzed using the PROTEAN^®^ IEF Cell.

### In-Gel Digestion

Protein spots were cut out and washed with a destaining buffer containing 50% CH_3_CN, 25 mM NH_4_HCO_3_ until CBB was completely removed. The gel spots were completely dehydrated with 100% CH_3_CN and dried using a Micro Vac (Tomy, Tokyo, Japan). Protein digestion was carried out with 10 µg/ml trypsin solution (Promega) in 50 mM NH_4_HCO_3_ for 16 h at 37 °C. Gel pieces were extracted twice with 50% CH_3_CN, 5% HCOOH. Each extract was combined and dried to 5 µl in the Micro Vac. One µl of 30% CH_3_CN, 0.6% HCOOH was added for MS analysis.

### Identification of Lipid Droplet Proteins by Liquid Chromatography–Tandem Mass Spectrometry

Separation and sequencing of tryptic peptides with liquid chromatography–tandem mass spectrometry (LC-MS/MS) was performed using a Q-Tof Premier (Jasco International) coupled with nanoACQUITY UPLC® (Waters). Peptide fragments were applied onto a nanoACQUITY BEH C18 100 µm × 100 mm column, and eluted at a flow rate of 0.4 µl/min for 30 min using a 3–40% linear gradient of solvent B of 0.1% HCOOH in CH_3_CN and 60–97% linear gradient of solvent A of 0.1% HCOOH in water. Analysis was performed using a positive ion mode at 3 kV capillary voltage. The mass range was set from 350 to 1700 m/z, and the MS/MS spectra were acquired for the peaks with at least 15 counts. The spectra were processed using ProteinLynx v4.1 software (Waters) and MASCOT (www.matrixscience.com) database searches of the NCBInr database for all Viridiplantae sequences. Peptides that could not be identified in the database were identified as partial sequences by *de novo* sequences using PepSeq (Waters). In this case, homologous proteins were predicted using BPASTP (DDBJ, http://www.ddbj.nig.ac.jp/welcome-j.html). Proteins were identified by the assignment of at least three peptide fragments, but proteins of less than 21 kDa were assigned using at least two peptide fragments. Proteins were predicted with homology of more than 70% using at least three peptides.

### Preparation of Antibodies Against Oleosin

Sample II was recovered as described in “Isolation and purification of lipid droplets” of Sect. 2.2. A five-fold volume of acetone was added to sample II. The sample was left at − 30 °C overnight and was centrifuged at 9000×*g* for 30 min. The supernatant was removed, and the precipitate was collected, and dried under reduced pressure. The dried samples were dissolved in 2 × SDS sample buffer for SDS-PAGE. The samples were applied for SDS-PAGE. Bands corresponding to oleosin of 15-kDa ~ 17-kDa were cut out, and the gel slices were pressed through a plastic syringe and stirred in 50 mM Tris-HCl buffer (pH 8.1) containing 0.1% SDS, 5% 2-mercaptoethanol, 1 mM EDTA overnight at room temperature. The sample was centrifuged at 9000×*g* for 20 min. The supernatant was recovered, and a five-fold volume of acetone was added to the supernatant. The sample was left at − 30 °C overnight and centrifuged at 9000×*g* for 30 min. The supernatant was removed, and the precipitate was collected and dried under reduced pressure. The dried sample was dissolved in 10 mM phosphate buffer containing 0.15 M NaCl, pH 7.1 (PBS) and used as purified oleosin for immunization. Purified oleosin (5 µg) was suspended in 250 µl PBS, mixed with the same amount of Freund’s complete adjuvant for the first injection or Freund’s incomplete adjuvant (Difco Lab) for subsequent injections. These mixtures were used as antigens for the preparation of polyclonal antibodies. SPF BALB/c mice were immunized by injecting antigens subcutaneously and intraperitoneally. Four injections at intervals of 10 days were given, and the mice were bled after the last injection. These experiments were approved by the Institutional Animal Care and Use Committee of Kinki University. Control serum was withdrawn from mice before the first injection.

### Preparation of Frozen Sections or Native Lipid Droplet and Immune-Confocal Microscopy

Sesame seeds were fixed in 4% formaldehyde for 8 h at 4 °C. After removal of formaldehyde, 10% sucrose solution was added. Samples were incubated for 4 h at 4 °C. The sucrose concentration was changed from 10 to 15%, and samples were incubated for 4 h at 4 °C. Afterward, the sucrose concentration was changed from 15 to 20%, and samples were incubated for 8 h at 4 °C. Samples were embedded in the OCT compound (Sakura Finetek) and cut into 2 µm thick frozen section using a Leica CM1850. Sections were incubated in 2% bovine serum albumen (BSA)/PBS, for 45 min at RT. Polyclonal mouse anti-sesame oleosin antibodies were diluted 100-fold with PBS and applied onto the sections for 2 h at RT. Sections were washed twice with PBS at intervals of 5 min. Fluorescein isothiocyanate (FITC)-conjugated goat anti-mouse IgG as the secondary antibody was diluted 100-fold with PBS and applied onto the sections for 2 h at RT. The sections were washed twice with PBS at intervals of 5 min. Next, a polyclonal anti-soybean 11S globulin antibody was diluted 100-fold with PBS and applied onto sections for 2 h at RT. The sections were washed twice with PBS at intervals of 5 min. Rhodamine-conjugated goat anti-rabbit IgG was diluted 100-fold with PBS and applied onto sections for 2 h at RT. The sections were washed twice with PBS at intervals of 5 min. All sections were observed using a confocal microscope (BioRad Radiance 2000).

Native lipid droplets as described in the “Isolation and purification of lipid droplets” in the Materials and Methods were prepared, and they were treated for 2 h at RT with four kinds of detergents, Triton X-100, Tween 20, SDS or CTAB, at 0.5% final concentrations. After washing with PBS, the samples were incubated with a polyclonal anti-soybean 11S globulin antibody for 2 h on ice, and washed with PBS. The samples were incubated with FITC-conjugated goat anti-rabbit IgG for 2 h on ice. After washing with PBS, the samples were observed with a BioRad confocal microscope (Radiance 2100).

## Results

### Isolation and Purification of Lipid Droplets

**Fig. 2:**
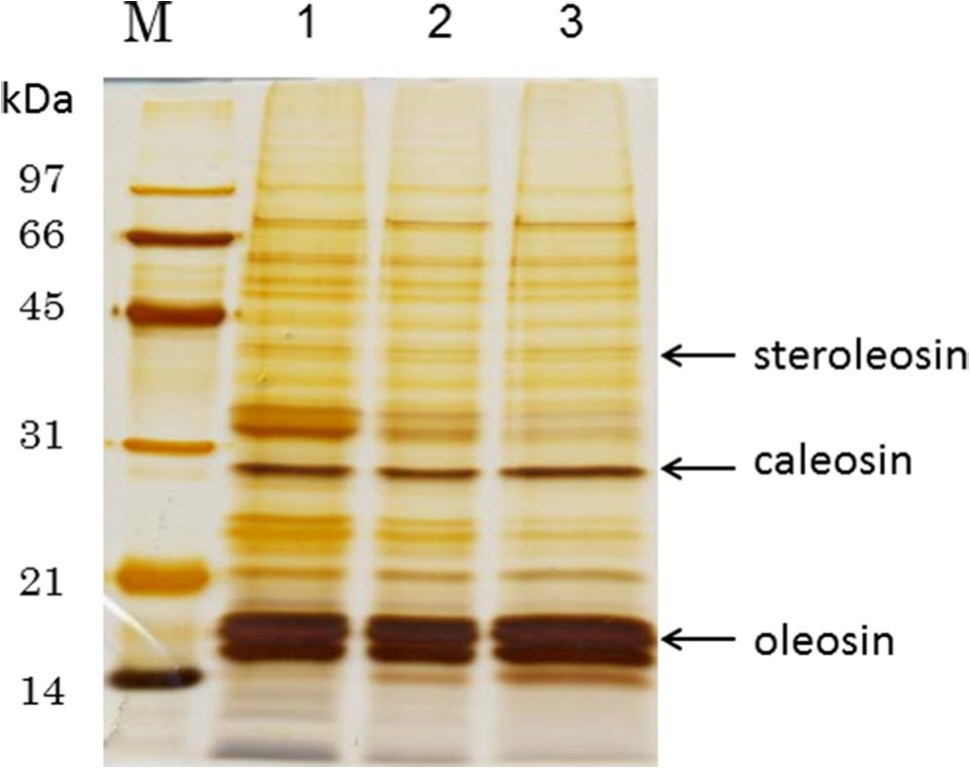
SDS-PAGE of lipid droplet samples from each step of the sucrose gradient centrifugation. Lane M shows the positions of molecular mass markers in kilodaltons (kDa). Lane 1, sample I; lane 2, sample II; lane 3, sample III, as described in the Materials and Methods. Arrows show the positions of oleosin, caleosin, and steroleosin

In this study, we attempted to isolate as many proteins from lipid droplets as possible, including proteins that weakly adhere to the outer monolayer surface of lipid droplets via ionic bonds. As a first step, the homogenizing conditions of sesame seeds were studied. After immersing in 0.6 M sucrose, 10 mM Na_2_HPO_4_–KH_2_PO_4_, pH 7.5 (homogenizing buffer), sesame seeds were homogenized with a polytron homogenizer PT2100 at constant speed from 15,000 to 22,000 rpm for 20 s. At less than 19,000 rpm, the yield of lipid droplet proteins was only one-tenth of that at higher speeds. There was no difference in the recovery amount of lipid droplet proteins from 20,000 to 22,000 rpm with the polytron homogenizer. Addition of 0.2 M KCl, 2 mM MgCl_2_, or 2 mM CaCl_2_ into the homogenizing buffer did not produce any effects on the yield of lipid droplets. However, the appearance of a few spots by two-dimensional electrophoresis became stronger with these salt additions, whereas that of a few spots became weaker (data not shown). Moreover, sesame lipid droplets were prepared in mild conditions without treatment with high salt concentrations or detergents [10] to isolate as many proteins from lipid droplets as possible. Sesame lipid droplets were purified through each step by sucrose gradient centrifugation after the isolation of lipid droplets (Fig. 2). The strengths of protein bands of oleosin, caleosin, and steroleosin, which have been reported as major components of lipid droplets [11], were not subjected to changes through each step.

### Identification of Lipid Droplet Proteins by LC-MS/MS

**Fig. 3:**
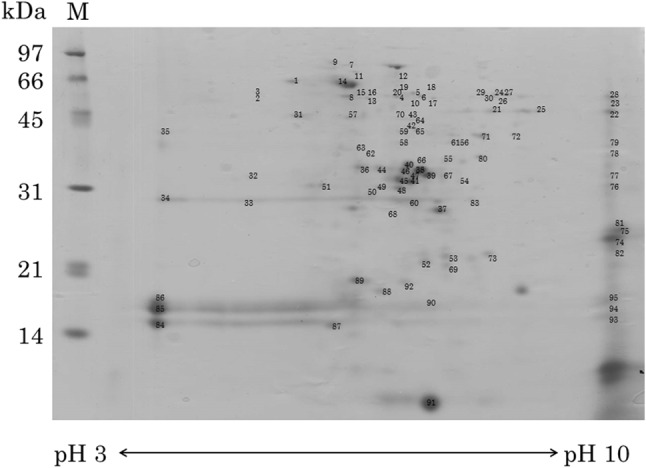
Two-dimensional electrophoresis of lipid droplet proteins in sample III from sesame seeds. Protein spots were identified using a Q-Tof Premier. The number of each spot corresponds to that in Table 1

Lipid droplet sample III was prepared for lipid droplet proteomics, and subjected to two-dimensional electrophoresis. As a result, 139 spots appeared on the two-dimensional electrophoresis gel as lipid droplet components (Fig. 3). Of 139 spots, 103 spots were identified by the assignment of at least three peptide fragments. The 103 proteins in lipid droplets were a remarkably high proportion of lipid droplet components, compared with the three major proteins of lipid droplets, oleosin, caleosin, and steroleosin, as reported previously [11]. In other plant lipid droplets, more than 20 proteins were identified in *Zea mays* [12] and more than 200 proteins in *Chlamydomonas reinhardtii* [13]. The number of proteins associated with lipid droplets increases with time [14, 15]. It has been suggested that OBAP1, a member of a discovered family of lipid droplet-associated proteins (OBAP) is involved in the regulation of lipid droplet size [16].

Results with LC-MS/MS showed that 103 spots were identified as lipid droplet proteins (Table 1), among them protein disulfide isomerase, calreticulin, BiP, and elongation factor were marker proteins of the endoplasmic reticulum (ER), and 11S globulin, 7S globulin, 2S albumin were marker proteins of protein bodies. Mitochondrial ATP synthase, mitochondrial processing peptidase, and 34-kDa outer mitochondrial membrane protein porin-like protein are mitochondrial origin proteins.

**Table 1:** Protein assignments of lipid droplet proteins by Q-T of analysis

Spot no.	Acc. no.	Protein name	Species	MW (kDa)/	pI (T)	MW (kDa)/	pI(E)	Match
Known oil body proteins								
No33	gi:6478218[NCBI]	Caleosin	*S. indicum*	27.78/	5.59	29/	4.5	7
No34	gi:6478218[NCBI]	Caleosin	*S. indicum*	27.78/	5.59	29/	3	6
No35	gi:21311775[NCBI]	Steroleosin-B	*S. indicum*	41.28/	6.99	41/	3	6
No63	gi:15824408[NCBI]	Steroleosin	*S. indicum*	39.71/	5.71	39/	6	7
No84	Q2TM28[EMBL]	Oleosin	*C. canephora*	15.17/	8.11	15/	3	3
No85	gi:10834827[NCBI]	Oleosin	*S. indicum*	17.41/	9.84	17/	3	4
No86	Q9XHP2[EMBL]	15kD oleosin	*S. indicum*	15.18/	10.08	17/	3	2
No93	Q2TM28[EMBL]	Oleosin	*C. canephora*	15.17/	8.11	15/	10	3
No94	gi:10834827[NCBI]	Oleosin	*S. indicum*	17.41/	9.84	17/	10	3
No95	Q9XHP2[EMBL]	15kD oleosin	*S. indicum*	15.18/	10.08	17/	10	4
Seed storage proteins								
No21	gi:13183177[NCBI]	7S globulin (homologue)	*S. indicum*	67.88/	7.55	47/	8	6
No22	gi:13183177[NCBI]	7S globulin	*S. indicum*	67.88/	7.55	47/	10	8
No25	gi:13183177[NCBI]	7S globulin (homologue)	*S. indicum*	67.88/	7.55	47/	8.8	4
No26	gi:75315270[NCBI]	11S globulin seed storage protein 2 precursor	*S. indicum*	52.08/	7.72	52/	8.2	11
No27	gi:13183177[NCBI]	7S globulin	*S. indicum*	67.88/	7.55	57/	8.2	7
No28	gi:13183177[NCBI]	7S globulin	*S. indicum*	67.88/	7.55	56/	10	4
No30	gi:75315270[NCBI]	11S globulin seed storage protein 2 precursor	*S. indicum*	52.08/	7.72	52/	7.9	14
No38	gi:81238592[NCBI]	11S globulin precursor isoform 3 (A subunit)	*S. indicum*	55.59/	8.23	33/	7	11
No39	gi:81238594[NCBI]	11S globulin precursor isoform 4 (A subunit)	*S. indicum*	52.99/	8.2	33/	7.2	17
No41	gi:75315270[NCBI]	11S globulin seed storage protein 2 precursor (A subunit)	*S. indicum*	52.08/	7.72	32/	6.9	15
No44	gi:13183173[NCBI]	11S globulin (A subunit)	*S. indicum*	56.78/	8.57	34/	6.4	8
No45	gi:75315270[NCBI]	11S globulin seed storage protein 2 precursor (A subunit)	*S. indicum*	52.08/	7.72	32/	6.7	13
No46	gi:81238592[NCBI]	11S globulin precursor isoform 3 (A subunit)	*S. indicum*	55.59/	8.23	33/	6.8	10
No47	gi:81238594[NCBI]	11S globulin precursor isoform 4 (A subunit)	*S. indicum*	52.99/	8.2	33/	6.9	8
No52	gi:81238594[NCBI]	11S globulin precursor isoform 4	*S. indicum*	52.99/	8.2	22/	7.1	5
No53	gi:81238594[NCBI]	11S globulin precursor isoform 4	*S. indicum*	52.99/	8.2	23/	7.4	4
No54	gi:81238594[NCBI]	11S globulin precursor isoform 4 (A subunit)	*S. indicum*	52.99/	8.2	33/	7.6	7
No55	gi:13183177[NCBI]	7S globulin	*S. indicum*	67.88/	7.55	35/	7.4	3
No60	gi:75315270[NCBI]	11S globulin seed storage protein 2 precursor (A subunit)	*S. indicum*	52.08/	7.72	29/	6.8	6
No66	gi:81238594[NCBI]	11S globulin precursor isoform 4 (A subunit) (homologue)	*S. indicum*	52.99/	8.2	36/	7.1	6
No67	gi:81238594[NCBI]	11S globulin precursor isoform 4 (A subunit)	*S. indicum*	52.99/	8.2	33/	7.4	7
No68	gi:81238594[NCBI]	11S globulin precursor isoform 4	*S. indicum*	52.99/	8.2	27/	6.5	6
No69	gi:81238594[NCBI]	11S globulin precursor isoform 4 (A subunit)	*S. indicum*	52.99/	8.2	21/	7.4	6
No74	gi:81238592[NCBI]	11S globulin precursor isoform 3 (B subunit)	*S. indicum*	55.59/	8.23	24/	10	7
No74	gi:81238594[NCBI]	11S globulin precursor isoform 4 (B subunit)	*S. indicum*	52.99/	8.2	24/	10	6
No75	gi:81238594[NCBI]	11S globulin precursor isoform 4 (B subunit)	*S. indicum*	52.99/	8.2	25/	10	4
No75	gi:75315270[NCBI]	11S globulin seed storage protein 2 precursor (B subunit)	*S. indicum*	52.08/	7.72	25/	10	7
No76	gi:75315270[NCBI]	11S globulin seed storage protein 2 precursor (A subunit)	*S. indicum*	51.79/	7.72	35/	10	12
No77	gi:81238592[NCBI]	11S globulin precursor isoform 3 (A subunit)	*S. indicum*	55.59/	8.23	33/	10	12
No77	gi:81238594[NCBI]	11S globulin precursor isoform 4 (A subunit)	*S. indicum*	52.99/	8.2	33/	10	8
No77	gi:13183173[NCBI]	11S globulin (A subunit)	*S. indicum*	56.78/	8.57	33/	10	6
No81	gi:75315270[NCBI]	11S globulin seed storage protein 2 precursor (B subunit)	*S. indicum*	52.08/	7.72	26/	10	10
No82	gi:13183173[NCBI]	11S globulin (B subunit)	*S. indicum*	56.78/	8.57	24/	10	10
No90	gi:81238592[NCBI]	11S globulin precursor isoform 3	*S. indicum*	55.59/	8.23	17/	7.5	3
No91	gi:75267546[NCBI]	2S seed storage protein 1 precursor(large subunit)	*S. indicum*	18.08/	6.1	9/	7.2	3
Membrane traffic proteins								
No1	Q9SRG3[Swiss-Prot]	Protein disulfide-isomerase 2 precursor	*A. thaliana*	56.36/	4.9	62/	5.2	3
No2	gi:117165712[NCBI]	Calreticulin-1	*G. max*	48.31/	4.43	58/	4.7	5
No7	gi:562006[NCBI]	PsHSP71.2	*P. sativum*	71.52/	5.17	80/	5.9	8
No9	Q587K1[EMBL]	BiP	*G. max*	73.59/	5.06	82/	5.7	4
No10	gi:14334534[NCBI]	Putative mitochondrial processing peptidase alpha subunit	*A. thaliana*	54.21/	6.16	49/	6.8	3
No11	gi:399940[NCBI]	Heat shock 70 kDa protein, mitochondrial precursor	*P. vulgaris*	72.72/	5.95	75/	6	8
No14	gi:16221[NCBI]	Chaperonin hsp60	*A. thaliana*	61.65/	5.66	60/	5.8	14
No15	gi:2501850[NCBI]	GDP dissociation inhibitor	*N. tabacum *	50.12/	5.44	57/	6.1	3
No16	gi:13959067[NCBI]	Mitochondrial processing peptidase	*A. marina*	59.37/	5.93	57/	6.2	4
No31	Q9S9N1[EMBL]	Heat shock protein hsp70	*A. thaliana*	70.91/	5.3	45/	5.2	3
No78	Q2PYX4[EMBL]	34kD outer mitochondrial membrane protein porin-like	*S. tuberosum*	29.42/	6.5	37/	10	4
No88	Q01545[Swiss-Prot]	18.8kDa class II heat shock protein	*I. nil*	18.77/	5.21	17/	6.4	3
No89	Q9SWE4[EMBL]	Low molecular weight heat-shock protein	*N. tabacum*	17.17/	5.59	19/	6	3
Cellular respiration proteins								
No4	gi:19281[NCBI]	Enolase	*L. esculentum*	47.76/	5.68	54/	6.7	5
No6	gi:19281[NCBI]	Enolase	*L. esculentum*	47.76/	5.68	53/	7	3
No42	gi:121485004[NCBI]	Cytosolic phosphoglycerate kinase	*H. annuus*	42.33/	5.82	42/	7	3
No43	gi:7159004[NCBI]	Alcohol dehydrogenase 2	*P. sinjiangenesis*	22.62/	5.28	44/	7	3
No56	gi:120676[NCBI]	Glyceraldehyde-3-phosphate dehydrogenase, cytosolic	*N. tabacum*	35.68/	6.14	39/	7.6	3
No58	gi:18202485[NCBI]	Malate dehydrogenase cytoplasmic	*Z. mays *	35.90/	5.77	40/	6.8	5
No59	Q655T1[EMBL]	Cytosolic phosphoglycerate kinase 1	*O. sativa*	42.27/	6.19	42/	6.8	4
No65	Q9FR11[EMBL]	Pyruvate dehydrogenase	*S. lycopersicum*	43.37/	8.06	41/	7.2	3
No71	gi:218157[NCBI]	Cytoplasmic aldolase	*O. sativa*	38.71/	6.56	40/	7.9	3
No72	Q43359[EMBL]	cytosolic glyceraldehyde-3-phosphate dehydrogenase GAPC4	*Z. mays*	36.45/	6.61	40/	8.4	4
Redox proteins								
No12	gi:15240075[NCBI]	Succinate dehydrogenase 1-1	*A. thaliana*	70.24/	5.86	67/	6.7	7
No18	gi:3309269[NCBI]	Ferric leghemoglobin reductase-2 precursor	*G. max*	53.31/	6.9	58/	7.2	3
No24	Q48561[Swiss-Prot]	Catalase-4	*G. max*	56.73/	6.8	56/	8.1	3
No29	gi:90818818[NCBI]	Catalase	*P. deltoides*	57.19/	7.09	56/	7.8	10
No36	Q9FZ42[Swiss-Prot]	Glucose and ribitol dehydrogenase homologue 1	*A. thaliana*	31.38/	6.11	34/	6.1	3
No37	gi:19423862[NCBI]	1 cys peroxiredoxin	*X. viscosa*	24.48/	6.31	27/	7.3	4
No61	gi:151301848[NCBI]	Putative aldo/keto reductase 2	*S. miltiorrhiza*	37.85/	6.09	40/	7.5	6
No65	Q6V4H0[EMBL]	10-hydroxygeraniol oxidoreductase	*C. roseus*	38.93/	6.67	41/	7.2	3
No70	gi:45935133[NCBI]	Putative dihydroflavonol reductase	*I. trifida*	46.11/	5.78	44/	6.8	4
Energy production proteins								
No5	gi:12986[NCBI]	F1 ATPase adenosinetriphosphatase	*H. annuus*	55.45/	6.02	55/	6.9	6
No17	gi:114408[NCBI]	ATP synthase subunit alpha, mitochondrial	*O. biennis*	55.84/	6.23	47/	7.2	3
No19	gi:5305369[NCBI]	ATP synthase alpha chain	*V. radiata*	55.55/	6.23	58/	6.7	4
No20	gi:903732[NCBI]	F1ATPase alpha subunit	*H. annuus*	55.76/	6.23	57/	6.6	10
Seed maturation proteins								
No32	Q9SEL0[EMBL]	Seed maturation protein PM24	*G. max*	26.84/	5.14	34/	4.6	4
No51	Q9SEL0[EMBL]	Seed maturation protein PM24	*G. max*	26.84/	5.14	32/	5.6	4
No87	gi:1141784[NCBI]	Em protein	*V. radiata*	10.93/	6.62	15/	5.7	4
No92	A6N8C4[EMBL]	Pathogen-related protein STH-2	*S. miltiorrhiza*	17.97/	5.4	19/	6.8	4
Protein synthesis related proteins								
No23	gi:24371057[NCBI]	Eukaryotic elongation factor 1A	*S. japonica*	49.74/	9.2	52/	10	4
No33	gi:124484511[NCBI]	Alpha chain of nascent polypeptide associated complex	*N. thamiana*	21.91/	4.32	29/	4.5	3
No62	gi:38350579[NCBI]	Cystein synthase	*N. plumbaginifolia*	34.12/	5.71	37/	6.2	4
No64	gi:11181616[NCBI]	Translational elongation factor EF-TuM	*Z. mays*	48.74/	5.99	43/	7.2	5
Other proteins								
No3	gi:1161252[NCBI]	Nucleosome assembly protein 1	*G. max*	41.07/	4.32	59/	4.7	3
No13	gi:37953301[NCBI]	Alanine aminotransferase	*C. annuus*	53.33/	5.29	50/	6.2	3
No37	gi:13183179[NCBI]	Cystatin	*S. indicum*	22.33/	6.17	27/	7.3	6
No40	Q8GSE7[EMBL]	TAG factor protein	*L. angustifolius*	32.21/	6.33	36/	6.8	5
No48	Q8GXX2[EMBL]	Putative uncharacterized protein	*A. thaliana*	26.70/	5.79	32/	6.7	3
No49	Q1PE68[EMBL]	hypothetical protein	*A. thaliana*	27.74/	7.64	32/	6.4	3
No50	Q49407[EMBL]	Putative uncharacterized protein AT4g18920	*A. thaliana*	28.67/	7.65	32/	6.3	3
No57	gi:122891673[NCBI]	Actin	*S. dulcis*	27.37/	5.21	44/	5.9	7
No70	gi:147774828[NCBI]	Hypothetical protein	*V. vinifera*	38.16/	6.32	44/	6.8	5
No73	Q8L9C2[EMBL]	Ethylene-responsive protein	*A. thaliana*	21.38/	5.53	23/	8	4

Spot no. corresponds to that in Fig. 3. Acc. no: accession number from NCBI, EMBL or Swiss-Prot database; MW(kDa)/pI(T): molecular weight(kDa)/isoelectric point of theoretical values from database; MW(kDa)/pI(E): molecular weight(kDa)/isoelectric point of experimental values; Match, number of matched peptides

### Localization of 11S Globulin in Lipid Droplets

**Fig. 4:**
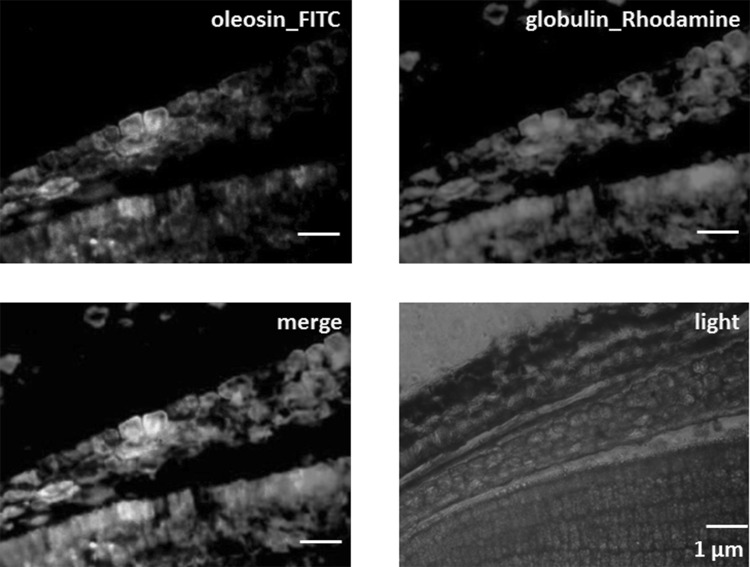
Localization of 11S globulins in lipid droplets of frozen sections. Frozen sections were incubated with polyclonal mouse anti-oleosin or polyclonal rabbit anti-soybean 11S globulin primary antibodies. After the anti-oleosin primary antibody, the sections were incubated with FITC-conjugated goat anti-mouse IgG secondary antibodies. Next, after the anti-soybean 11S globulin primary antibody, the sections were incubated with rhodamine-conjugated goat anti-rabbit IgG secondary antibody. Scale bars show 1 µm

**Fig. 5:**
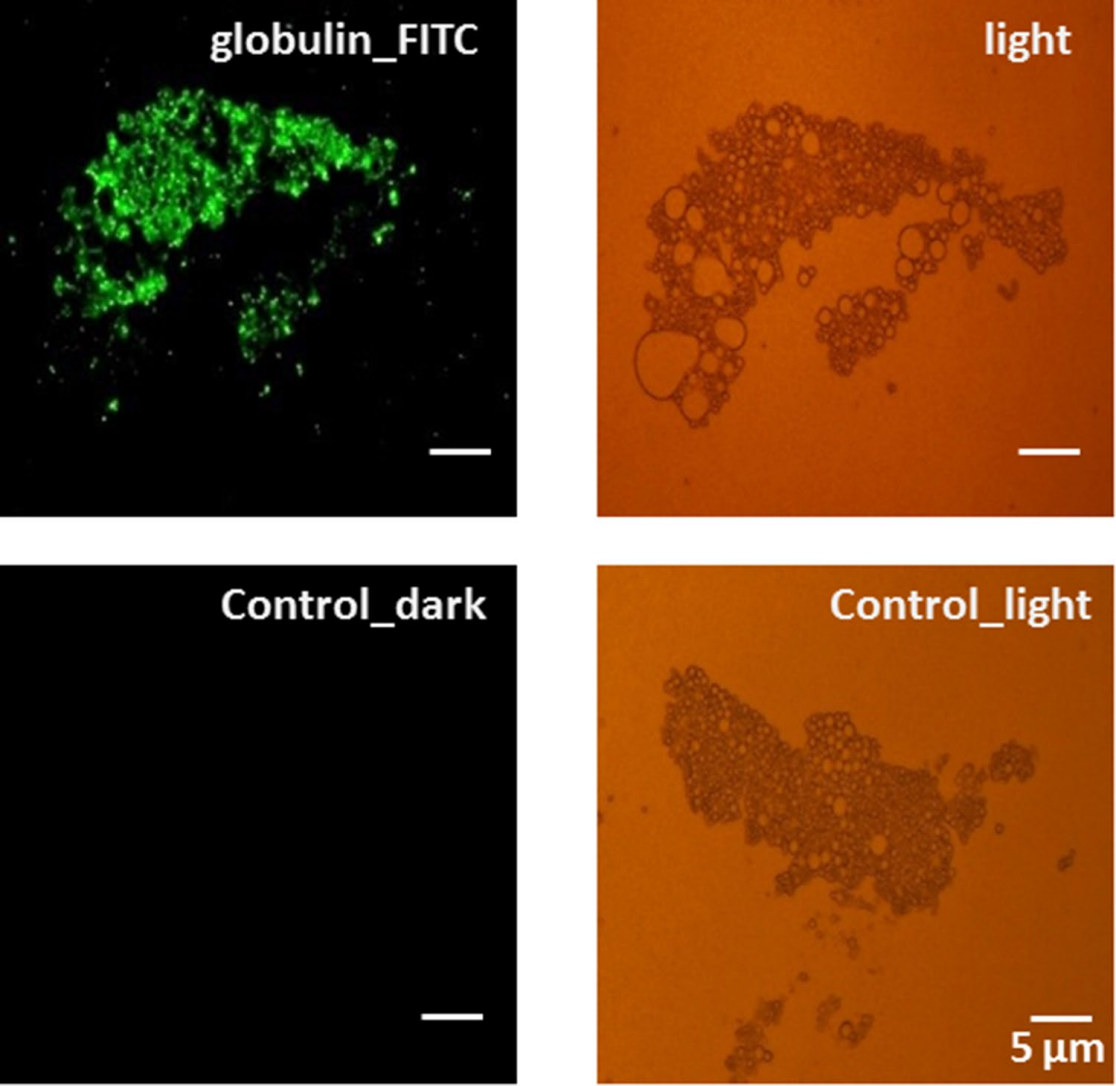
Localization of 11S globulins in native lipid droplets. Native lipid droplets, isolated as described in “Isolation and purification of lipid droplets” in the Materials and Methods were prepared to study the localization of 11S globulins. Native lipid droplets treated with CTAB were incubated with polyclonal rabbit anti-soybean 11 S globulin as the primary antibody and with FITC-conjugated goat anti rabbit IgG as the secondary antibody. Scale bars show 5 µm

The localization of 11S globulin in lipid droplets was shown using frozen sections and immunoconfocal microscopy (Fig. 4). When lipid droplets were confirmed by using antibody against oleosin, the fluorescence from 11S globulin was observed in the lipid droplets. Moreover, isolated native lipid droplets were treated with four kinds of detergents, as described in the Materials and Methods. Only the lipid droplets treated with CTAB emitted fluorescence, when anti-soybean 11S globulin antibody as the primary antibody and FITC-conjugated goat anti rabbit IgG as the secondary antibody were applied (Fig. 5). The lipid droplets treated with other detergents, Triton X-100, Tween 20, or SDS, did not emit any fluorescence. The difference in results between CTAB and the three other detergents might be due to the curious properties of lipid droplet membranes.

### Modification of Lipid Droplet Proteins

**Fig. 6:**
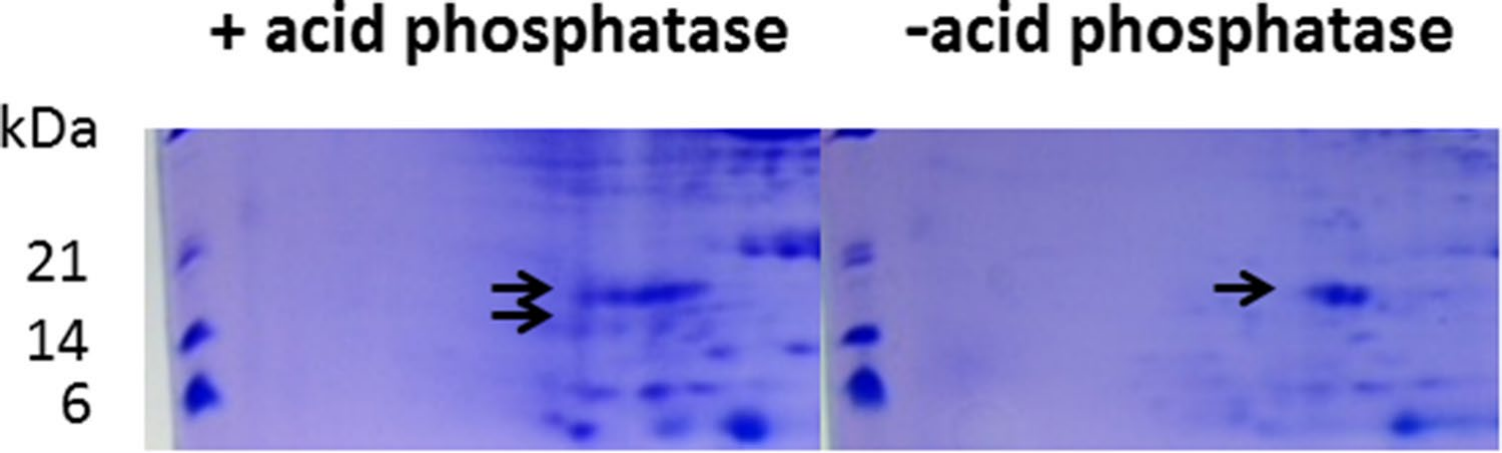
Two-dimensional electrophoresis of sample II treated with acid phosphatase. The positions of oleosin proteins are shown by an arrow. Left panel (with acid phosphatase), right panel (without acid phosphatase)

Lipid droplets are surrounded by the monolayer, which might have negative electric charges on the surface, from the ER membrane. This suggested the existence of phosphate groups on the surface of lipid droplets (cf. Fig. 6). Modification of several proteins, including oleosin, was observed (cf. Fig. 3). Modification of oleosin was investigated by phosphatase treatment. Acid phosphatase treatment was much more effective in shifting from acidic to basic sites (Fig. 6). The results clearly indicated that oleosin proteins are phosphorylated. It was conjectured that phosphate groups might prevent the fusion of lipid droplets, although it had been believed that oleosin proteins play an important role in inhibiting lipid droplets from adhering to each other ([10], trypsin treatment exp).

## Discussion

Although 103 spots were identified as lipid droplet proteins (Table 1), protein disulfide isomerase, calreticulin, BiP, and elongation factor were used as marker proteins of the ER, and 11S globulin, 7S globulin, 2S albumin were used as marker proteins for protein bodies. Mitochondrial ATP synthase, mitochondrial processing peptidase, and 34-kDa outer mitochondrial membrane protein porin-like protein are marker proteins for mitochondria. These results suggested that interactions between lipid droplets and other organelles, including the ER, protein bodies, and mitochondria, might occur through attachment between membranes [17]. The results were not due to organelle degradation, as indicated by the fact that each protein from the other organelles, other than the protein body, was derived from the organelle membrane. However, enolase, alcohol dehydrogenase, malate dehydrogenase, aldolase, phosphoglycerate kinase, and glyceraldehyde-3-phosphate dehydrogenase are cytosolic proteins, and they might be contaminants at the process of lipid droplet isolation. Indeed, Katavic et al. [18] described that hydrophobic internal protein body membranes might adhere to lipid droplets, which makes it very difficult to isolate lipid droplets without contamination of storage proteins even after stringent washing with salt and urea. Some papers suggested about the possibilities of contamination from the other organelles during the isolation of lipid droplets [19–21]. Alternatively, they might be correlated with the formation of lipid droplets, as shown in Figs. 4 and 5, indicating that lipid droplets indeed consist of 11S globulins. The existence of 103 spots in lipid droplets suggested that sesame lipid droplets do not only function in the storage of sesame oil, but also might dynamically function through interactions with other organelles [22, 23].

The existence of heat shock proteins in lipid droplets is important in the transportation of proteins. Three kinds of 70-kDa heat shock protein, one 60-kDa heat shock protein, and two kinds of low molecular weight, 17-kDa and 18-kDa, heat shock proteins were detected as lipid droplet proteins. It is thought that the three major proteins, oleosin, caleosin, and steroleosin, are coincidentally transported to lipid droplets in the formation of lipid droplets from the ER [4]. It is necessary to reconsider the transportation of lipid droplet components. Total proteins from the ER as well as lipid droplets were also investigated by LC-MS/MS. However, oleosin, caleosin, and steroleosin proteins were not confirmed in the ER (data not shown), although the storage proteins 7S globulin, 11S globulin, and 2S albumin were detected in the ER.

Six spots of 7S globulin, 27 spots of 11S globulin, and one spot of 2S albumin, which are authentically localized in protein bodies, were identified as lipid droplet proteins in this study. Although all six spots of 7S globulin were a mature type, 24 of 27 spots in 11S globulin were precursor types (Fig. 3; Table 1). These suggested that 11S globulins were processed and transported from the ER to protein bodies after synthesis in the ER as 11S globulin precursors, are different from 7S globulins. Although both 7 and 11 S globulins are synthesized as precursor types in the ER, processed during transportation, and finally stored as mature types in protein bodies [24], differences between 7 and 11S globulins were observed at the transportation and processing steps. The differences might reflect their retention times in the ER. The retention time of 11S globulin precursors in the ER might be longer than that of 7S globulin precursors, because 11S globulins are formed with disulfide bonds, whereas 7S globulins do not have disulfide bonds. Although we cannot explain the reason why differences between 11S globulin and 7S globulin precursors occurred at the processing and transportation steps from the ER to protein bodies, it is conceivable that 7S globulin precursors and 11S globulin precursors are transported through different processing steps to protein bodies to prevent mixing between 7S globulin precursors and 11S globulin precursors [25, 26].

What is the process of 11S globulin precursor accumulation in lipid droplets? There are two possibilities. One is 11S globulin precursors are incorporated into lipid droplets during transportation from the ER to protein bodies after synthesis in the ER as 11S globulin precursors. However, considering that 11S globulins are transported directly from the ER to the Golgi apparatus, it is difficult to consider this possibility. The second is that 11S globulin precursors are incorporated into lipid droplets while lipid droplets are being formed. For the formation of lipid droplets, three hypotheses were considered (Fig. 1). (1) Lipid droplets bud off toward the cytosol (Fig. 1a). (2) Lipid droplets are formed and leave behind a gap in the ER membrane, and afterwards the gap closes immediately (Fig. 1b). In both a and b, when triacylglycerols (TAGs) accumulate between the leaflets of the ER membrane and a critical size is reached, lipid droplets might bud off from the ER membrane of the leaflet of the cytosol side (Fig. 1a) or lipid droplets might be excised from the ER membrane of the leaflets of the cytosol side and lumen side (Fig. 1b). (3) The formation of TAG-filled secretory vesicles undergo remodeling of the ER-derived bilayer to yield a monolayer from the ER membrane of the leaflet on the cytosol side, covering lipid droplets. The ER membrane from the leaflet of the lumen side is enfolded in the lipid droplet (Fig. 1c). From the point of view that 24 of 27 spots in 11S globulin were precursor types (Table 1) and that they were inside the lipid droplets (Fig. 5), lipid droplets are likely to be formed as in the model of Fig. 1c.

Furthermore, the model in Fig. 1c made it possible to explain the fact that protein disulfide isomerase, calreticulin, BiP, and elongation factor, as marker proteins of the ER, were found in lipid droplets.

## Abbreviation

CBB: Coomassie brilliant blue
CTAB: Cetyl trimethyl ammonium bromide
ER: Endoplasmic reticulum
FITC: Fluorescein isothiocyanate
LC-MS/MS: Liquid chromatography-tandem mass spectrometry
PBS: Phosphate buffered saline
SDS-PAGE: Sodium dodecyl sulfate-polyacrylamide gel electrophoresis
TAG: Triacylglycerol
